# Factors Associated with Job Satisfaction among University Teachers in Northeastern Region of China: A Cross-Sectional Study

**DOI:** 10.3390/ijerph121012761

**Published:** 2015-10-14

**Authors:** Bochen Pan, Xue Shen, Li Liu, Yilong Yang, Lie Wang

**Affiliations:** 1 Center for Assisted Reproduction, Department of Obstetrics and Gynecology, Shengjing Hospital, China Medical University, No 36 Sanhao Street, Heping District, Shenyang 110004, China; E-Mail: panbc@sj-hospital.org; 2 Department of Social Medicine, School of Public Health, China Medical University, No.77 Puhe Road, Shenyang North New District, Shenyang 110122, China; E-Mails: xueshen89@163.com (X.S.); liul@mail.cmu.edu.cn (L.L.); yyylll390@163.com (Y.Y.)

**Keywords:** job satisfaction, perceived organizational support, psychological capital, occupational stress, university teachers

## Abstract

***Objective*:** Teachers’ job satisfaction is one of the key factors in institutional dynamics and is generally considered to be the primary variable by which the effectiveness of an organization’s human resource is evaluated. The objectives of this study were to assess the level of job satisfaction among university teachers and to clarify the associated factors. ***Method*:** A cross-sectional study was conducted between November 2013 and January 2014. Teachers from six universities in Shenyang, China were randomly sampled. The job satisfaction scale Minnesota Satisfaction Questionnaire (MSQ), perceived organizational support (POS), psychological capital questionnaire (PCQ-24), and effort-reward imbalance scale (ERI) together with questions about demographic and working factors were administered in questionnaires distributed to 1500 university teachers. Hierarchical linear regression analyses were performed to explore the related factors. ***Results*:** 1210 effective responses were obtained (effective respondent rate 80.7%). The average score of overall job satisfaction was 69.71. Hierarchical linear regression analysis revealed that turnover intention, occupational stress and chronic disease all had negative impacts on job satisfaction, whereas perceived organizational support, psychological capital and higher monthly income were positively associated with job satisfaction among the university teachers. Age was also linked to the level of job satisfaction. All the variables explained 60.7% of the variance in job satisfaction. ***Conclusions*:** Chinese university teachers had a moderate level of job satisfaction. Demographic and working characteristics were associated factors for job satisfaction. Perceived organizational support showed the strongest association with job satisfaction. Results of the study indicate that improving the perceived organizational support may increase the level of job satisfaction for university teachers.

## 1. Introduction

It is generally believed that a teacher, considered as an engineer of the human soul, plays an important role in society. They are considered pillars of society because they shoulder the responsibility of educating and training students upon whom our future relies. Previous research has revealed that employees who are satisfied with their job are more likely to be creative, innovative and initiate the breakthroughs that can increase their job performance [1]. On the other hand, a teacher who is dissatisfied with his or her job may become irritable and tense which may bring inefficiency and other negative effects to the students’ learning process. 

A study performed in the UK showed that university academic staff rated conducting research and time constraints as the main causes of stress at work, and 74.1% and 14.7% of the teachers had moderate to serious levels of stress, respectively [2]. A similar survey conducted in an Australian university showed that of all the staff members, academic staff who engaged in both teaching and research had the highest psychological distress and lowest job satisfaction, indicating that high work loads may be the cause for work stress [3]. The situation in China could be worse. Due to the rapid development of higher education and expansion of enrollment in universities, competition is becoming increasingly intense between colleges or universities. As a result, Chinese university teachers nowadays are confronted with increased work loads and may feel significant pressure from teaching, research, publishing academic papers and professional status evaluations. Recent research has revealed that university teachers in China are exposed to a high level of occupational stress and many of them suffer from depressive symptoms [4,5]. All the above data seem to suggest that Chinese university teachers are particularly susceptible to heavy physical and mental health burdens, which in turn may induce their dissatisfaction with work.

Job satisfaction can be defined as a pleasurable or positive emotional state resulting from the appraisal of one’s job or job experiences [6]. Job satisfaction includes intrinsic and extrinsic dimensions. The intrinsic job satisfaction refers to how people feel about the nature of the job tasks themselves such as work activity, ability utilization, sense of achievement, while the extrinsic job satisfaction refers to how people feel about aspects of the working situation that are external to the job tasks or work itself such as working policies, human relations and work compensation [7]. As a global and multidimensional construct, job satisfaction has received considerable attention by researchers, predominantly focusing on employees in business, companies and hospitals [8,9,10,11]. However, few investigations pertaining to the factors associated with job satisfaction among Chinese university teachers have been conducted. Some studies suggested that gender, salary, occupational stress, years of teaching experience and organizational justice were the factors influencing job satisfaction [12,13,14,15]. Empirical studies in China also showed that job stress, salary, leadership management, organizational climate, evaluation orientation and academic field were related to teachers’ job satisfaction [16,17]. Moreover, previous studies demonstrated that job satisfaction was significantly related to job performance, quality of life, stress, burnout, anxiety and turnover intention [18,19,20,21,22]. In the present study, we focused on both the intrinsic and the extrinsic aspects of job satisfaction.

With increasing recognition of the value of positive organizational behavior, organizations seek to improve employees’ job satisfaction by strengthening the psychosocial resources of employees. Perceived organizational support (POS) reflects the degree to which employees believe that their work organization values their contribution and cares about their well-being [23]. POS is conducive to increasing the employees` affective attachment to the organization and their expectancy that greater efforts toward meeting organizational goals will be rewarded [23]. As an important concept in organizational theory, POS has been reported to be positively related to job satisfaction, job performance, organizational justice, and affective commitment [24,25]. For Chinese university faculty members, it was reported that more POS was associated with better job performance [24]. POS was also reported to be associated with organizational citizenship behavior and turnover intentions among Chinese employees [26].

Psychological capital (PsyCap) has been defined as an individual’s positive psychological state of development [27]. As a high-order construct of positive organizational behavior, PsyCap comprises four state-like psychological resources: self-efficacy, hope, optimism and resilience [28]. Few studies about PsyCap in Chinese university teachers have been reported. Previous research in other populations has shown that PsyCap is positively associated with job performance, job satisfaction and well-being [28,29]. In addition, PsyCap is considered as a positive resource for combating burnout, stress and depressive symptoms [30,31]. Thus, employees with higher levels of PsyCap may be more satisfied with their jobs.

POS, PsyCap and occupational stress are key variables in the research on job satisfaction. While POS and PsyCap are considered as positive associated factors [24,27], occupational stress is regarded as a negative associated factor [4]. Additionally, as POS and occupational stress are two constructs at the organizational level [23], they could be recognized as external associated factors. On the other hand, PsyCap could be considered as an internal factor because it refers to the psychological capacity of an individual [28,32]. Previous research has mainly focused on the impacts of individual variables (POS, PsyCap or occupational stress) on job satisfaction, whereas the integrated effects of the three variables have seldom been reported, especially in university teachers.

The objectives of the present study were to evaluate the level of job satisfaction among Chinese university teachers and to explore its associated factors. Based on the previous reports, demographic characteristics (gender, age, marital status, educational levels, chronic disease and physical exercise), working conditions (professional position, monthly income and turnover intention), and occupational psychosocial factors including occupational stress, POS and PsyCap were investigated to determine the associated factors of job satisfaction among Chinese university teachers. The present study would not only explore the integrated effects of both positive and negative factors on job satisfaction, but also the impacts of internal and external factors on job satisfaction after adjusting for demographic characteristics and working conditions. 

## 2. Materials and Method

### 2.1. Research Design and Sample

A cross-sectional survey among university teachers was conducted from November 2013 to January 2014 in Shenyang, the capital city of Liaoning province and the center of higher education in Northeast China. According to Shenyang Statistical information Net in 2013 [33], Universities of Liaoning province are mainly located in Shenyang, with the total number of full-time teachers reaching 24,888. For this study, six universities (two comprehensive universities and four universities specializing in medicine and architecture) were selected and 25% of the full-time teachers were randomly sampled from each involved university. 

Anonymous self-administered questionnaires were distributed to 1500 teachers after obtaining their written informed consent. After eliminating unqualified questionnaires, a total of 1210 effective responses were obtained (effective response rate: 80.7%). The study was approved by the Committee on Human Experimentation of China Medical University, and the study procedures were in accordance with ethical standards.

### 2.2. Demographic and Working Characteristics

Demographic and working characteristics collected included gender, age, marital status, educational levels, professional positions, monthly income, physical exercise, turnover intention and chronic disease. Marital status was categorized as single/widowed/divorced and married/cohabiting. Educational levels were divided into bachelor/master/doctor. Professional positions were classified into assistant/lecturer/associate professor/professor. Monthly income (RMB) was divided into <4000 yuan, 4000–6999 yuan, and ≥7000 yuan groups. Participants were categorized as having chronic disease if they responded “yes” to ever receiving a diagnosis of any listed diseases such as hypertension, diabetes, gout, cardiovascular disease, chronic gastritis or back pain. Turnover intention was judged to be present if participants answered “yes” to the question, “Do you consider leaving the current institution?” Physical exercise (doing exercise at least once every week) was classified into “no” and “yes”.

### 2.3. Measurement of Job Satisfaction

Job satisfaction was measured with the short Chinese version of the Minnesota Satisfaction Questionnaire (MSQ) [34]. The 20-item questionnaire includes two dimensions: intrinsic job satisfaction and extrinsic job satisfaction, with each item being rated on a 5-point Likert-type scale, ranging from 1 (very dissatisfied) to 5 (very satisfied). Intrinsic job satisfaction includes 12 items (e.g., “Being able to keep busy all the time”), and extrinsic job satisfaction includes 6 items (e.g., “The way company policies are put into practice”). Higher scores reflect a higher level of job satisfaction. The overall satisfaction is indicated by the sum score of all the 20 items which ranges from 20 to 100. A score of 60 indicates neutral attitude, a score ranging from 61 to 79 indicates being moderately satisfied, and a score of 80 indicates being highly satisfied [35]. The Cronbach’s alpha coefficients for the overall job satisfaction, intrinsic job satisfaction, and extrinsic job satisfaction were 0.964, 0.899 and 0.947, respectively. 

### 2.4. Measurement of Perceived Organizational Support (POS)

POS was assessed by using the 9-item Chinese version of the Survey of Perceived Organizational Support (SPOS) [23,36]. Respondents indicated the extent of their agreement with each statement on a 7-point Likert-type scale, ranging from 1 (strongly disagree) to 7 (strongly agree), with higher values indicating higher levels of POS. One example item is: “the organization strongly considers my goals and values”. The coefficient alpha for this scale was 0.915.

### 2.5. Measurement of Occupational Stress

The Chinese version of the effort-reward imbalance (ERI) model was used to assess the occupational stress experienced by university teachers [37]. The 23-item ERI questionnaire is comprised of three subscales: effort (six items, e.g., “Do you have enough time to do everything”), reward (11 items, e.g., “How satisfied are you with your usual take-home pay?”) and overcommitment (6 items, e.g., “People close to me say I sacrifice myself too much for my job”). Responses to the “effort” and “reward” items are scored on a five-point scale (from completely disagree to couldn’t agree more), with 1 reflecting no stressful experience and 5 reflecting very stressful experience for effort and higher scores indicating agreeable experience for the reward. For the overcommitment subscale, responses are scored from 1 to 4, with higher scores indicating stronger (over) commitment to work. Effort-reward ratio (ERR) is calculated by a predefined algorithm to quantify the degree mismatch between high cost and low gain, with a correction factor of 0.5454 [38]. If the value of ERR ratio is beyond 1.0, it indicates one’s reward is not met with the effort he or she has spent. In this study, the Cronbach’s alpha coefficient for the effort, reward and overcommitment subscales was 0.927, 0.907 and 0.802, respectively.

### 2.6. Measurement of Psychological Capital

The level of PsyCap was evaluated with the Chinese version of the 24-item Psychological Capital Questionnaire (PCQ) [28]. The PCQ is comprised of four dimensions: self efficacy (e.g., “I am confident helping to set targets/goals in my work area.”), hope (e.g., “I can think of many ways to reach my current work goals.”), resilience (e.g., “I usually take stressful things at work in stride.”) and optimism (e.g., “When things are uncertain for me at work I usually expect the best.”). Each of the four dimensions includes six items, ranging from 1 (strongly disagree) to 6 (strongly agree). Higher scores indicate higher levels of experienced psychological capital. The PCQ has been widely used, and demonstrates adequate reliability and validity in multiple samples [28]. For the total scale, the Cronbach’s alpha coefficient was 0.949. Cronbach’s alpha coefficients for self efficacy, hope, resilience and optimism were 0.905, 0.904, 0.818 and 0.745, respectively.

### 2.7. Statistical Analysis

The distributions of job satisfaction in categorical variables were examined by the Student’s t-test and one-way ANOVA. Pearson’s correlation analysis was used to assess the correlations between study variables. Hierarchical linear regression analyses were performed to explore the factors associated with job satisfaction. For the regression model, job satisfaction was set as the dependent variable. In Block 1 of the regression analyses, the demographic and working characteristics were put in the model. In Block 2, ERI, PsyCap and POS were added. Variances of job satisfaction explained by different variable groups were examined by ∆R^2^. All study variables were standardized before analyses to account for differences in all scale scores. All analyses were performed using SPSS 17.0 (SPSS China Corp, Shanghai, China) for Windows. Statistical significance was defined as *p* < 0.05 (two-tailed).

## 3. Results

### 3.1. Participant Characteristics

The basic characteristics of the participants and the mean scores of job satisfaction in the demographic categories are shown in Table 1. Among the 1,210 participants, 513 (42.4%) were men and 697(57.6%) were women. Men reported significantly higher score of job satisfaction than women (*p* = 0.001). Participants whose age ranged from 31 to 50 showed a lower level of job satisfaction than other groups (*p* < 0.001). Marital status also had significant influence on job satisfaction. Married or cohabiting teachers had lower levels of job satisfaction than those who lived alone (*p* = 0.005). Different professional positions showed a different level of job satisfaction. Teaching assistants and professors reported significantly higher levels of job satisfaction than lecturers and associate professors (*p* < 0.001). Doing exercise regularly, educational levels and monthly income were shown to be positively associated with job satisfaction (*p* < 0.001). In addition, participants with turnover intention or chronic disease had significantly lower levels of job satisfaction (*p* < 0.001). Occupational stress (ERR > 1) was observed in 22.3% of the participants and these teachers had markedly lower scores than those with ERR ≤ 1 in job satisfaction. 

**Table 1:** Participants’ characteristics and differences in job satisfaction.

Variables	Number	%	Job Satisfaction (Mean ± SD)	*p*
**Gender**				
Males	513	42.4	71.29 ± 15.15	0.001
Females	697	57.6	68.54 ± 14.10	
**Age**				
≤30	174	14.4	73.64 ± 13.33	<0.001
31–40	565	46.7	67.67 ± 13.81	
41–50	367	30.3	70.58 ± 15.90	
>50	104	8.6	71.11 ± 14.55	
**Marital status**				
Single/widow/separated	186	15.4	72.49 ± 14.69	0.005
Married/cohabitation	1024	84.6	69.20 ± 14.55	
**Educational level**				
Bachelor	168	13.9	67.21 ± 14.06	<0.001
Master	557	46.0	68.34 ± 13.84	
Doctor	485	40.1	72.14 ± 15.32	
**Professional positions**				
Assistant	105	8.7	72.07 ± 14.16	<0.001
Lecturer	493	40.7	68.79 ± 13.93	
Associate professor	445	36.8	68.14 ± 14.59	
Professor	167	13.8	75.10 ± 15.60	
**Monthly income (yuan, RMB)**				
<4000	295	24.4	66.87 ± 14.64	<0.001
4000–6999	733	60.6	68.51 ± 13.90	
≥7000	182	15	79.12 ± 13.74	
**Physical exercise**				
No	447	36.9	67.22 ± 14.94	<0.001
Yes	763	63.1	71.16 ± 14.23	
**Turnover intention**				
No	826	68.3	73.30 ± 13.94	<0.001
Yes	384	31.7	61.97 ± 12.95	
**Chronic disease**				
No	739	61.1	72.08 ± 14.68	<0.001
Yes	471	38.9	65.98 ± 13.72	
ERR				
≤1	940	77.7	72.68 ± 13.38	<0.001
>1	270	22.3	59.35 ± 14.01	

ERR: effort/reward ratio.

### 3.2. Correlations of Continuous Independent Variables with Job Satisfaction

The means, standard deviations (SD), and correlations of continuous variables with job satisfaction are presented in Table 2. The average age of the participants was 39.15 (SD = 8.02) years old. POS and PsyCap were positively correlated with job satisfaction, whereas ERR and overcommitment were negatively correlated with job satisfaction.

**Table 2:** Means, standard deviations (SD), and correlations of continuous variables with job satisfaction.

Variables	Mean	SD	Correlation with Job Satisfaction
1. Age	39.15	8.02	0.028
2. POS	4.67	1.26	0.715 **
3. PsyCap	4.26	0.78	0.650 **
4. ERR	0.77	0.49	−0.528 **
5. Overcommitment	14.42	3.71	−0.427 **

POS: perceived organizational support; ERR: effort–reward ratio; PsyCap: psychological capital. ** *p* < 0.01 (two-tailed).

### 3.3. Factors Associated with Overall Job Satisfaction

The results of hierarchical linear regression analysis for exploring the major factors associated with overall job satisfaction are presented in Table 3. In Block 1, age, monthly income, turnover intention, chronic disease and physical exercise were significantly associated with job satisfaction, explaining 20.5% of the variance in overall job satisfaction. In Block 2, the level of job satisfaction was significantly associated with POS, PsyCap, ERR, and overcommitment, explaining an additional 40.0% of the variance in job satisfaction. However, gender and education had no significant association with job satisfaction. The contribution of all impact factors to the model R-square was 60.7%.

### 3.4. Factors Associated with Intrinsic Job Satisfaction

The results of the hierarchical linear regression analysis for the intrinsic job satisfaction are presented in Table 3. In Block 1, the demographic and working characteristics explained 18.0% of the variance. In Block 2, POS, PsyCap, ERR and overcommitment made up an additional 37.8% of the variance in job satisfaction. However, overcommitment was not significantly associated with job satisfaction (*p* > 0.05). All the dependent variables accounted for 56.0% of the variance in intrinsic job satisfaction.

### 3.5. Factors Associated with Extrinsic Job Satisfaction

The major factors associated with extrinsic job satisfaction are shown in Table 3. In Block 1, the demographic and working characteristics accounted for 21.5% of the variance. In Block 2, POS, PsyCap, ERR and overcommitment explained an additional 37.6% of the variance in job satisfaction. All variables explained 59.2% of the variance in extrinsic job satisfaction.

**Table 3:** Hierarchical linear regression analyses for exploring associated factors for job satisfaction.

Variables	Overall Job Satisfaction		Intrinsic Job Satisfaction		Extrinsic Job Satisfaction		
	Block 1 (β)	Block 2 (β)	Block 1 (β)	Block 2 (β)	Block 1 (β)	Block 2 (β)	
Gender	−0.034	−0.019	−0.027	−0.014	−0.042	−0.027	
Age	−0.145 **	−0.066 *	−0.140 **	−0.067 *	−0.142 **	−0.059 *	
Marital status	−0.023	0.039 *	-0.018	0.040	−0.030	0.033	
Educational level 1 (Master *vs.* Bachelor)	0.020	0.017	0.014	0.011	0.030	0.030	
Educational level 2 (Doctor *vs.* Bachelor)	0.054	0.038	0.061	0.041	0.049	0.042	
Monthly income 1	0.080 *	0.003	0.083 *	0.011	0.078 *	0.002	
Monthly income 2	0.304 **	0.100 **	0.302 **	0.105 **	0.281 **	0.083 **	
Turnover intention (yes *vs.* no)	−0.294 **	−0.045 *	−0.270 **	−0.034	−0.307 **	−0.058 **	
Chronic disease (yes *vs.* no)	−0.084 **	0.023	−0.069 *	0.032	−0.109 **	0.000	
Physical exercise (≥1 times/week *vs.* no)	0.060 *	0.019	0.053	0.013	0.063 *	0.021	
POS		0.414 **		0.361 **		0.477 **	
PsyCap		0.279 **		0.325 **		0.167 **	
ERR		−0.128 **		−0.113 **		−0.136 **	
Overcommitment		−0.058 *		−0.050		-0.075 **	
F	32.254 **	134.528 **	27.563 **	110.787 **	34.086 **	126.548 **	
Adjust *R^2^*	0.205	0.607	0.180	0.560	0.215	0.592	
∆R^2^	0.212 **	0.400 **	0.187 **	0.378 **	0.221 **	0.376 **	

POS: perceived organizational support; PsyCap: psychological capital; ERR: effort–reward ratio. Marital status: married/cohabiting *vs.* single/divorced/widowed/separated; Monthly income1: 4000-6999 yuan RMB *vs.* <4000 yuan RMB; Monthly income2: ≥7000 yuan RMB *vs.* <4000 yuan RMB. * *p* < 0.05, ** *p* < 0.01 (two-tailed).

## 4. Discussion

The present research investigated the level of job satisfaction among university teachers in the northeastern region of China and explored its associated factors. Although job satisfaction in university teachers has received increasing attention worldwide, the relevant research is still under represented in China. Our results revealed that Chinese university teachers enjoyed a moderate level of satisfaction (the average score of job satisfaction was 69.71, SD = 14.61), which was at the same level of teachers in special needs schools of South Africa (69.55) [39], lower than an American standardized group [39], but higher than Chinese community health workers (68.2) [19] and doctors (65.86) [10]. Similar findings were reported for university teachers in other areas of China [16,40].

As for the risk factors, both ERR and overcommitment were found to have a negative association with job satisfaction, which is consistent with previous findings [19,37,41]. In addition, we found that 22.3% of the participants experienced occupational stress (ERR > 1). In China, the transformation of the education system has produced an immense workload for university teachers. They are required not only to provide knowledge and quality-oriented education for students, but also to do research, obtain project grants and publish papers. Furthermore, they are under the burden of promotion, performance appraisal, and redundancy. Occupational stress has been considered as a major hazard for employees as it may affect job satisfaction, job performance and quality of life and can even induce psychological problems such as burnout, depression and anxiety [14,20,41,42,43,44]. Therefore, effective measures should be taken by university administrations to decrease occupational stress and improve job satisfaction among university teachers.

In this study, POS was found to be associated with overall job satisfaction among Chinese university teachers (the standardized regression coefficient was 0.414). Employees who perceive a high level of organizational support tend to feel confident and hopeful about their desired job goals and are able to have both the motivation and plans to achieve their goals. It should be noted that in our survey, the mean score (4.67) of POS in the institutions was only a little higher than the neutral value (4.0), even lower than the score of “to some extent agreed” (5.0), indicating the inadequacy of POS for the teachers in these institutions. It was reported that POS such as stress-reduction interventions perceived by university staff could increase their job satisfaction [45]. Therefore, providing more support to the university teachers by adopting employee (teacher)-centered approaches such as taking into account the employee’s best interests, valuing their work and providing help when they encounter difficulties may improve their job satisfaction.

PsyCap was also found to be positively associated with job satisfaction. Teachers with higher levels of PsyCap might have more confidence and exert greater effort to pursue success (self-efficacy), preserve the will to accomplish a teaching task or goal (hope), bounce back from adversity or failure with positive psychological capacity (resilience), and have positive expectations and attributes regarding outcomes (optimism) [27]. Thus, teachers with higher levels of PsyCap may be more satisfied with their jobs. Therefore, it may be helpful for the university administrators to invest resources to help teachers develop PsyCap so as to relieve their occupational stress and improve their job satisfaction.

The results also demonstrated that the positive factors (POS and PsyCap) exerted stronger effects on job satisfaction than the negative factor (occupational stress), and external factors played a more powerful role than the internal factor. Since POS are directly derived from the organizations and it represents the acceptance and respect for teachers` ability and value [23], it wouldn’t be difficult to understand that POS constituted the strongest associated factor of overall job satisfaction. In addition, POS, PsyCap and occupational stress as a whole were found to account for positive variance in both intrinsic job satisfaction (∆R^2^:37.8%) and extrinsic job satisfaction (∆R^2^:37.6%).

With respect to the demographics and working characteristics, reports have shown that job satisfaction in university teachers is related to gender, age, professional titles, salary, and years of teaching experience [11,21,46,47]. In the present study, we found that turnover intention and chronic disease were negatively associated with job satisfaction among Chinese university teachers. Teachers who sometimes or frequently had turnover intentions reported lower level of satisfaction in comparison with those who never had. Since chronic disease may cause deterioration in physical and mental health, it can be expected that teachers with chronic disease have a lower score in job satisfaction than those in good health. Physical exercise may be considered as an important factor for restoration of personal resources. In our study, physical exercise was found to be positively related to job satisfaction, which indicated that physical exercise might help relieve pressure, and make teachers more likely to get involved in and enjoy their work. Another factor of job satisfaction found in this study was monthly income. It is generally believed that teachers with a higher monthly income may be more satisfied with their jobs because it gives them the sense of achievement. In addition, university teachers, like other types of workers, tend to be satisfied if they feel their pay reflects their market value.

Finally, age was found to be associated with job satisfaction. It has been established that job satisfaction varies with age in different occupations. Some studies observed a “U” shaped relationship between age and job satisfaction [48], but others did not [49]. Our results were in accordance with the U shape relationship, with the 31 to 40 years old group having significantly lower level of job satisfaction than the other age groups. The effect of age was also reflected in the regression model. The explanation might be that the youngest teachers (≤30 years old) had joined the teaching faculty only recently. This was a period of time when the job content itself was the focus of their attention and also the period of time during which their career development was fast, so they could be content with their job accomplishment. On the other hand, experienced teachers might become more realistic and satisfied with their career achievements compared to younger colleagues. In contrast, the 31-40 years old teachers could be under more pressure from high workload or requirements as they had to do research, to apply for promotion, and to shoulder the burden of raising a family, all of which could contribute to the decrease in their job satisfaction.

Our study results are important because they provide university administrators with information on the associated factors which may be modified to help develop effective measures to improve job satisfaction. Measures such as establishing flexible work schedules for employees, acknowledging contribution, allocating enough funding for scientific research, increasing opportunities for career advancement and encouraging involvement of faculty in decision-making all may have the potential to increase teachers’ job satisfaction. Furthermore, our study may provide a new perspective for the university administrators. By considering the intrinsic satisfaction and extrinsic satisfaction separately, we are able to demonstrate the different contributions of the associated factors, which may have practical implications. For example, POS was shown to contribute more to the extrinsic satisfaction than PsyCap; this indicates that improving POS might be more effective for enhancing the extrinsic satisfaction than improving PsyCap. Likewise, this study provides a new integrated perspective so that university administrators could promote teachers’ job satisfaction by developing relevant positive factors. For example, as POS was found to be positively related to job satisfaction, the university administrators can seek to implement policies that enhance teachers’ well-being and value their contributions. Similarly, the university administrators should also be encouraged to integrate the positive psychological resources of self-efficacy, hope, optimism and resilience in their management to improve overall job satisfaction. For self-efficacy improvement, university administrators should provide more professional trainings or opportunities to the teachers. To increase hope, university administrators should encourage teachers to consider multiple pathways to achieve their goals. To enhance optimism amongst teachers, university administrators should help teachers obtain a positive attribution style towards success and failure. For resilience, university administrators should motivate teachers to seek more solutions in order to overcome obstacles [50,51,52]. In short, by adopting various measures to modify these associated factors, we would be able to increase teachers’ enthusiasm in work and job satisfaction.

This study has several limitations. First, it mainly relied on the self-report of the participants, which risks response bias on job satisfaction. Second, generalizations from our results may be weakened since the sample comprised only a small proportion of all teachers in China. Third, variables of physical exercise and turnover intention were measured with single items, which might compromise the validity of relevant results. Finally, due to the cross-sectional design, causal relationships between variables could not be reached. Future studies could incorporate a longitudinal design to increase the power of the findings.

## 5. Conclusions

In conclusion, our findings revealed that university teachers in the northeast of China enjoyed a moderate level of job satisfaction. Demographic and working characteristics were associated factors for job satisfaction. In particular, income was positively, whereas turnover intention was negatively, associated with job satisfaction, and there was a U shape relationship between age and job satisfaction. However, POS and PsyCap were the respective first and second most important positive contributing factors for job satisfaction while occupational stress (ERR) was the most influential negative factor. Therefore, attention should be focused on modifying these factors with the purpose of increasing job satisfaction among university teachers. 
